# Temperature Dependency of Insect’s Wingbeat Frequencies: An Empirical Approach to Temperature Correction

**DOI:** 10.3390/insects15050342

**Published:** 2024-05-10

**Authors:** Topu Saha, Adrien P. Genoud, Jung H. Park, Benjamin P. Thomas

**Affiliations:** 1 Department of Physics, New Jersey Institute of Technology, University Heights, Newark, NJ 07102, USA; ts627@njit.edu; 2 Institut Lumière Matière, UMR 5306, Université Claude Bernard Lyon 1, CNRS, F-69100 Villeurbanne, France; ap.genoud@gmail.com; 3 Department of Data Science, New Jersey Institute of Technology, University Heights, Newark, NJ 07102, USA; jp784@njit.edu

**Keywords:** wingbeat frequency, temperature, optical sensor, acoustic sensor, monitoring, clustering, environmental factors, field experiment

## Abstract

**Simple Summary:**

In this study, we explored how the wingbeat frequency of flying insects changes with air temperature. By analyzing over 300,000 insect observations using a near-infrared light sensor made during an eight-month field experiment, we observed that flying insects increase their wingbeat frequency when air temperature rises and that this trend was even more significant for insects with naturally higher wingbeat frequencies. Based on our findings, we developed a model to predict how wingbeat frequency typically changes with temperature. This model is designed to improve the precision of insect clustering, often used with entomological optical or acoustic sensors. Although it may not account for the unique responses of specific insect species to temperature variations, this empirical approach provides a general insight into the relationship between wingbeat frequency and temperature, which can be used to improve the effectiveness of insect classification from photonic or acoustic sensors.

**Abstract:**

This study examines the relationship between the wingbeat frequency of flying insects and ambient temperature, leveraging data from over 302,000 insect observations obtained using a near-infrared optical sensor during an eight-month field experiment. By measuring the wingbeat frequency as well as wing and body optical cross-sections of each insect in conjunction with the ambient temperature, we identified five clusters of insects and analyzed how their average wingbeat frequencies evolved over temperatures ranging from 10 °C to 38 °C. Our findings reveal a positive correlation between temperature and wingbeat frequency, with a more pronounced increase observed at higher wingbeat frequencies. Frequencies increased on average by 2.02 Hz/°C at 50 Hz, and up to 9.63 Hz/°C at 525 Hz, and a general model is proposed. This model offers a valuable tool for correcting wingbeat frequencies with temperature, enhancing the accuracy of insect clustering by optical and acoustic sensors. While this approach does not account for species-specific responses to temperature changes, our research provides a general insight, based on all species present during the field experiment, into the intricate dynamics of insect flight behavior in relation to environmental factors.

## 1. Introduction

Over the past decade, there have been notable interest and advancements in the development of new technology based on optical and acoustic sensors. These technologies try to address the overall lack of abundance data on insect populations [1,2] and hold potential for applications in agriculture, where pest monitoring and the decline of pollinators are critical, and in public health, for vector control strategies. Among these new methods are various optical sensors [3,4,5,6,7,8,9,10,11,12,13], including entomological lidars [7,9,14,15,16,17,18,19,20,21] and radars [22,23,24,25,26,27,28,29,30,31], as well as acoustic sensing [32,33,34]. Smart traps, imaging technologies, and computer vision [35,36,37,38,39,40,41,42] also offer alternative ways to track and evaluate insect abundance, while some instruments coupled these observations with lethal lasers to directly eliminate pests [43,44,45,46,47,48,49,50,51]. Often, the analysis of the data provided by these instruments uses machine learning tools to identify insects [7,15,32,52,53,54,55]. This approach has highlighted the wingbeat frequency as a significant predictor variable for identifying and discriminating among flying insect species.

While the core technologies often originate in physics, applied optics, and computer science, their applications are directly aimed at addressing pressing needs in entomology, specifically in the monitoring of insect abundance. Optical and acoustic sensors currently do not offer the same level of taxonomic resolution compared to traditional capture-based methods. However, as demonstrated in this article, they are capable of monitoring thousands of insects per day [4]. This capability significantly enhances the statistical power and temporal resolution of abundance measurements. They provide a critical trade-off by allowing for continuous, automated operation over extended periods, which drastically reduces labor requirements and associated costs. The challenge of achieving finer taxonomic resolution with these technologies is well recognized and is a subject of ongoing research within the entomological and technological communities. Efforts to refine taxonomic resolution include improving the clustering of insect species derived from the data collected by these instruments.

Wingbeat frequency, a parameter that plays a role in various biological and ecological functions, including mating and thermoregulation [56,57,58,59,60,61,62], is one of the main features used for clustering. The accurate interpretation and utilization of wingbeat frequency data are contingent upon a comprehensive understanding of its dependency on environmental factors, most notably temperature. In addition to being related to air density, temperature has been shown to influence insect muscle function and, by extension, wingbeat frequency [63]. Thus, this work contributes to the ongoing effort to refine the clustering of data collected by acoustic and optical sensors by providing a broad model of the temperature dependency of wingbeat frequency suited for large clusters of unknown species.

In light of these considerations, this contribution aimed to provide an empirical analysis of the temperature dependency of wingbeat frequency among flying insects and provide a general model for the correction of the wingbeat frequency of insects as a function of the air temperature. To achieve this, we conducted a comprehensive 8-month observational campaign in New Jersey, USA, utilizing a bistatic optical sensor to collect data on over 302,000 insect observations directly in the field [4]. This vast dataset includes detailed recordings of wingbeat frequencies, but also synchronized measurements of atmospheric conditions, specifically temperature and relative humidity, at the time of each observation. The relationship between wingbeat frequency and temperature has been explored in a few studies, albeit often within the context of a specific insect family, genus, or species, for example, with *Culicidae* [64,65], *Periplaneta* [66], *Diptera* and *Apidae* [67], *Coleopterida* [68], and *Lepidoptera* [69], to name a few. By analyzing data from the many insect species naturally present at the field location, this work offers a broader view of how temperature influences wingbeat frequency across a wide array of insect taxa. While this generalized approach may be less accurate for a specific species, this correction may enhance the clustering and discrimination of insects using wingbeat frequency, particularly in applications involving optical or acoustic sensors that suffer from low taxonomic resolution. 

## 2. Materials and Methods

### 2.1. Entomological Bistatic Optical Sensor System (eBoss)

**Figure 1:**
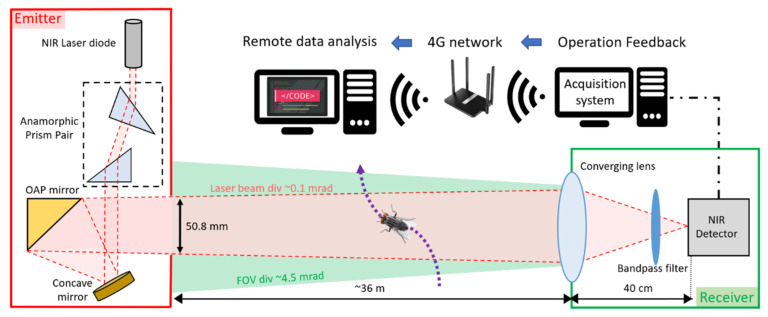
Optical layout of the eBoss instrument.

The optical sensor used in this study is named the Entomological Bistatic Optical Sensor System, or eBoss. It operates as a bistatic device, featuring a transmitter and a receiver that can be placed from 1 to 100 m apart, with a specific distance of 36 m in this current study. Figure 1 illustrates that the transmitter component houses a continuous near-infrared (NIR) laser diode (CPS980, Thorlabs, Newton, NJ, USA), which operates at a power of 5 mW and emits at a wavelength of 980 nm at 20 °C. The laser outputs an elliptical beam that is then shaped into a circular Gaussian beam via an anamorphic prism pair that has a 3× magnification factor. This beam is then expanded by being directed first towards a concave mirror and then reflected by an off-axis parabolic (OAP) gold mirror. The OAP mirror specifically reflects the beam’s central portion, with a diameter of 50.8 mm, filtering out the beam’s peripheral areas to achieve a consistent energy density along the beam’s path (also referred to as “flat top approximation”). The laser beam is maintained at a height ranging from 20 to 80 cm above the ground and is aimed at the receiver’s converging lens, which has an effective focal length of 40 cm. After passing through a spectral bandpass filter (950–1000 nm wavelength), the focused light reaches a silicon amplified photodetector (PDA36A2, Thorlabs, USA). The optical signal is captured at a sampling rate of 30,517 Hz by a 16-bit digitizer (M4i4420- × 8, Spectrum, Hackensack, NJ, USA) with a 3 V range. This data acquisition setup is linked to a standard desktop computer (Dell Technologies, Round Rock, TX, USA), which is connected to a 4G LTE router allowing for remote system monitoring. Additionally, a weather station (WS-1002-WIFI, Ambient Weather, Chandler, AZ, USA) is located approximately 15 m from the receiver to track weather conditions every minute. The eBoss was set up in a field in Secaucus (Hudson County, NJ, USA) bordered by a woodlot of roughly 1 hectare. Grass was mowed when necessary to prevent it from interfering with the laser beam. The device was deployed on 20 April and collected data until 21 December 2022.

### 2.2. Data Analysis

**Figure 2:**
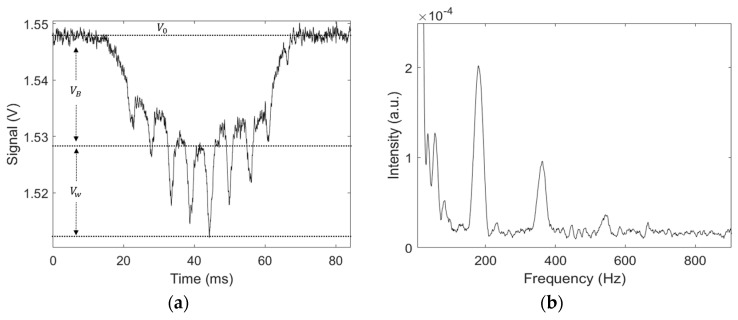
(**a**) An example of an optical signal caused by a flying insect; the dotted line at the top indicates the background signal, lower dotted lines represent the drop in intensity due to the insect’s body and wings, respectively. $V_{0}$ represents the background signal, while $V_{B}$ and $V_{w}$ represent the body and wing contributions to the measured voltage. (**b**) Frequency spectrum of the insect signal, showing the first and fundamental peak corresponding to the wingbeat frequency of the insect (~180 Hz); the following peaks are the harmonics.

The recorded signal represents the light intensity that reaches the detector located on the receiver end. As an insect passes through the laser beam, various parts of the insect, including its body and wings, scatter, diffract, and absorb a portion of the incoming light. This interaction causes a notable rise in optical extinction along the beam’s path, resulting in a decrease in the baseline signal level, often resembling a Gaussian shape. This reduction in the signal, known as a transit signal, is characterized by fluctuations in amplitude due to the rapid flapping of the insect’s wings. An illustration of this type of signal can be seen in Figure 2a. By performing a fast Fourier transform (FFT), the wingbeat frequency of the insect can be retrieved, as shown in Figure 2b.

Furthermore, by evaluating the relative variation of intensity due to both body and wings of the specimen, one can determine the optical extinction cross-sections for both wings and body. When no target is present in the laser beam, the detector’s voltage is labeled as the background signal $V_{0}$, as illustrated in Figure 2a. Meanwhile, the voltages recorded while an insect passes through are designated $V_{w}$ for the wings and $V_{B}$ for the body; see Figure 2a. With the cross-sectional area A of the laser beam known, the optical extinction cross-sections for the wings ($\sigma_{W}$) and body ($\sigma_{B}$) can be evaluated utilizing Equations (1a) and (1b):

(1a) $$\sigma_{w}=\frac{V_{0}-V_{w}}{V_{0}}\times A$$

(1b) $$\sigma_{B}=\frac{V_{0}-V_{B}}{V_{0}}\times A$$

Both $V_{W}$ and $V_{B}$ are evaluated by taking the average of the 10% minimum value. Finally, the wing-to-body cross-section ratio can be evaluated by the following Equation (2):

(2) $$\sigma_{w/b}=\frac{\sigma_{w}}{\sigma_{w}+\sigma_{B}}$$

The duration of a transit typically spans about 100 milliseconds, with occurrences shorter than 10 milliseconds being automatically excluded, while the lengthiest events may last up to 1 s. Transit events can also be triggered by non-insect entities: any non-transparent objects with an extinction cross-section larger than approximately a mm^2^ will result in a decrease in the signal; such objects are commonly falling leaves, large pollen grains, water droplets, birds, or any other objects intersecting the laser beam. To identify insect-specific events, a detection algorithm is employed that detects signal reductions beyond a specific threshold, utilizing wing patterns to distinguish insects from non-insect entities. This method has been extensively documented in earlier works [3]. During rainfall, water droplets produce transit signals that are identifiable from those of insects due to the lack of frequency components within the 10–900 Hz range. Such events are readily excluded during light rain, allowing for uninterrupted data collection. Nonetheless, during medium to heavy rain, the persistent influx of water droplets across the beam obstructs the identification of potential transits.

## 3. Results

**Figure 3:**
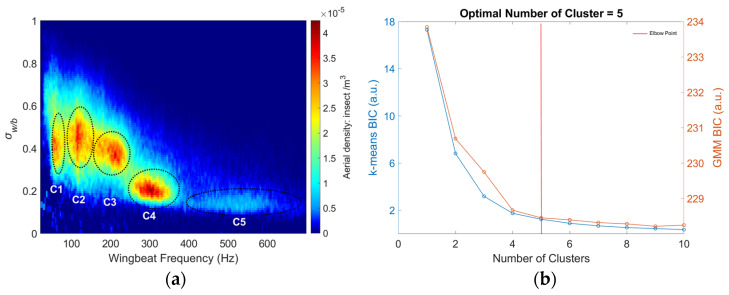
(**a**) The wingbeat frequency of each event as a function of the wing-to-body cross-section ratio, the five identified clusters are labeled C1 to C5. (**b**) BIC values for both GMM (ref line) and K-mean (blue line) methods as a function of the number of clusters, showing the ideal number of clusters at the elbow point.

During the eight-month period of the study, a total of 302,093 insect transit signals were recorded. Figure 3 depicts the distribution of insect transit signals based on their wingbeat frequency and their wing-to-body cross-section ratio. Within this distribution, several clusters become apparent. To analyze these clusters, a Gaussian mixture model (GMM) and K-means clustering were used independently to evaluate the optimal number of clusters. The Bayesian information criterion (BIC) was calculated for cluster counts ranging from 1 to 10. The results, shown in Figure 3b, include the BIC values for both the GMM and K-means methods. The optimal number of clusters was determined using the elbow method and identified using the methodology described in Raghavan et al. [70]. Both GMM and K-means are in agreement and the ideal number of cluster count was determined to be five. These clusters are highlighted in Figure 3a with black dashed circles.

The exact species composition of these clusters is unknown; however, both the wingbeat frequency and wing-to-body cross-section ratios may provide information about the potential species they include. The first cluster, located in the upper left section of the figure, comprises insects with relatively large wings in comparison to their bodies and low wingbeat frequencies, primarily including Lepidoptera (such as butterflies and moths) and Odonata (dragonflies and damselflies). Clusters two and three encompass insects whose wingbeat frequencies range from 80 Hz to 250 Hz and whose wing-to-body cross-section ratios fall between 0.25 and 0.65. These clusters are home to a variety of species from the orders *Diptera* (flies), *Hymenoptera* (bees and wasps), *Coleoptera* (beetles), *Orthoptera* (grasshoppers, crickets), and *Neuroptera* (lacewings). The two remaining clusters, four and five, correspond to mainly female and male mosquitoes, respectively. Mosquitoes are characterized by their high wingbeat frequencies, with females typically ranging between 250 and 400 Hz and males between 350 and 700 Hz. Although some small midges might also reach wingbeat frequencies over 250 Hz, their wing optical cross-sections are too small to be detected by the instrument, making their presence in Figure 3a unlikely.

**Figure 4:**
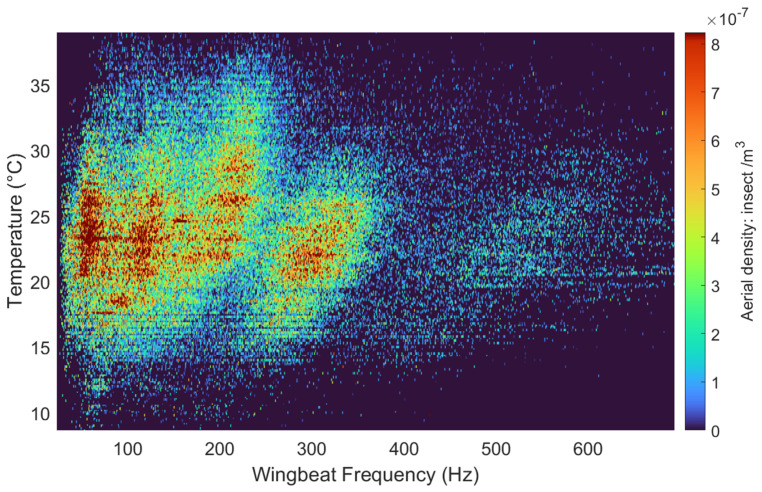
The wingbeat frequency of each event as a function of the temperature recorded at the time of the transit by the weather station.

Using the temperature data from the co-located weather station, the timestamp of each insect transit is used to retrieve the ambient temperature at the time of observation. Figure 4 presents transit events as a function of both wingbeat frequency and temperature, showing here again the five clusters visible in Figure 3a. 

**Figure 5:**
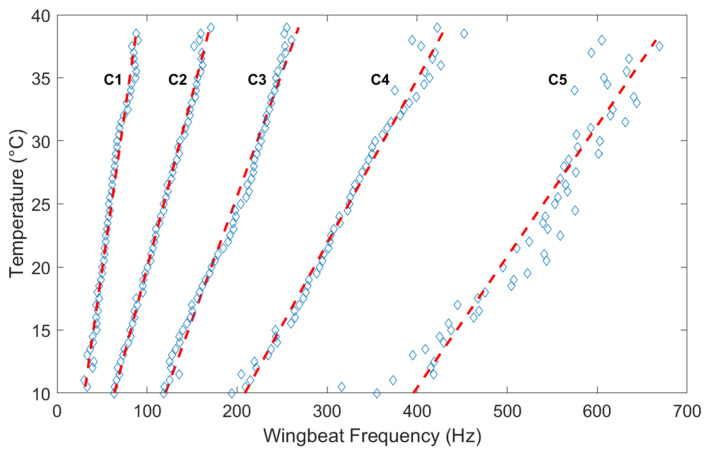
Cluster 1 to Cluster 5 show the average wingbeat frequency per temperature range (0.5 degree Celsius per bin) for each of the five clusters as well as a linear fit of the resulting points (red dashed lines).

For each cluster, the mean wingbeat frequency is computed for every interval of 0.5 degrees Celsius, with the outcomes subsequently modeled using a linear fit, as depicted in Figure 5. Due to the decreased activity of insects at lower temperatures, 95% of the transit occurrences are observed at temperatures exceeding 10 degrees Celsius [4]. Consequently, observations recorded below 10 degrees Celsius were omitted due to their averages being derived from a limited number of events, rendering them susceptible to significant statistical fluctuations. 

The slope (expressed in Hz/°C), retrieved from the linear fit of each cluster, and the average wingbeat frequency of the five clusters at 20 °C, are presented in Table 1. 

**Figure 6:**
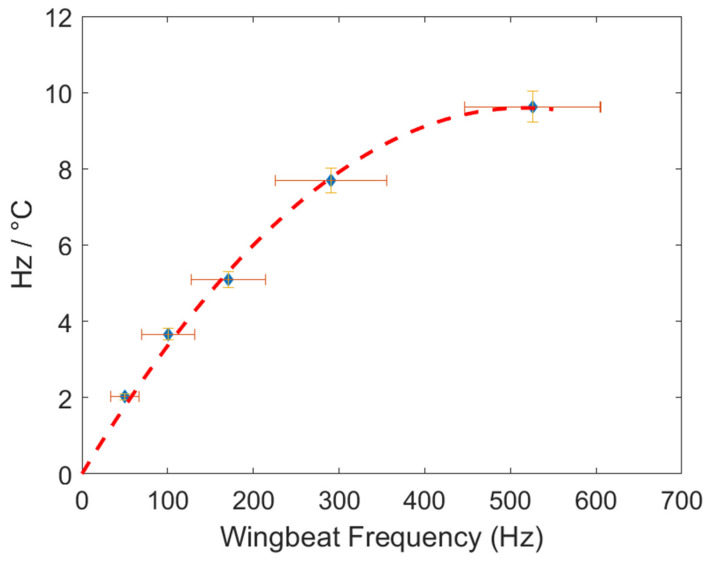
Second-degree polynomial fit of slope values obtained from each of the five individual clusters. The horizontal error bar represents the dispersion of wingbeat frequency, and the vertical error bar indicates the standard deviation of each slope value.

By using the slope values from each cluster as data points, a generalized temperature–frequency correction can be determined using a second-degree polynomial fit with $a=-3.605\times 10^{-5},b=0.0372,c=0,$ as shown in Figure 6 and Equation (3).

As a result, a general equation for temperature correction can be derived:

(3) $$f_{corrected}=f_{initial}+\left(-3.605\times 10^{-5}\times {{(f}_{initial})\;}^{2}+0.0372\times f_{initial}\right)\times \left(T_{ref}-T\right)\;$$

where $f_{corrected}$ is the wingbeat frequency after temperature correction, $f_{initial}$ is the wingbeat frequency measured by the optical instrument, $T_{ref}$ is the reference temperature, chosen as 20 °C for this study, and T is the air temperature in degrees Celsius, at which the insect flight observation is made and therefore at which $f_{initial}$ is determined.

**Figure 7:**
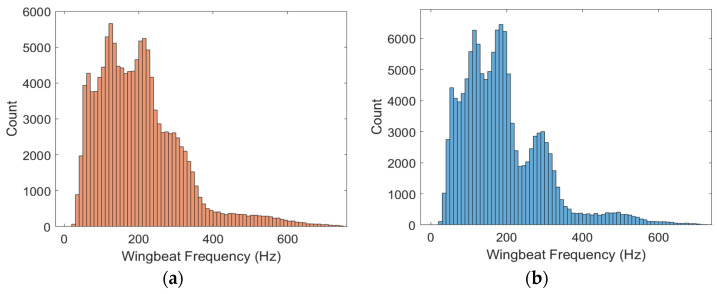
(**a**) Initial wingbeat frequency distribution; (**b**) wingbeat frequency distribution after correction for a reference temperature of 20 °C.

As an example of this empirical approach, the raw wingbeat frequency distribution obtained during this 8-month campaign is presented in Figure 7a, and the same distribution obtained after correction at 20 °C is presented in Figure 7b. 

## 4. Discussion and Conclusions

Our analysis focused on the variation in wingbeat frequency with temperature across multiple flying insect species. Using a near-infrared sensor, the wingbeat frequencies of more than 300,000 observed insects were retrieved over the course of eight months. Because the field campaign was conducted day and night from spring to winter 2022, wingbeat frequency measurements were made over a large range of temperatures, from −5 °C to 38 °C, although the analysis was conducted only between 10 °C and 38 °C as very few insects were flying below 10 °C. Signals were regrouped in clusters identified through a combination of wingbeat frequencies and the wing-to-body cross-section ratios, and the average wingbeat frequency of each cluster was studied as a function of the air temperature at the time the signals were measured. From this large dataset, we found that flying insects increase their wingbeat frequency linearly with increasing temperature and that this trend was more pronounced in insects with naturally higher wingbeat frequencies. 

Our results align well with findings from other studies in the field. Table 2 presents our findings compared to those of other studies: 

However, Unwin et al. [67] looked at other species, notably multiple species of bees and bumblebees, that led to a negative correlation with temperature. Similarly, Sotavalta in 1963 [73] found that the wingbeat frequencies of *A. mellifera* and *Bombus pascuorum* were independent of temperature. Parmezan et al. [74] also observed several wild bees and wasps and noticed a positive correlation between wingbeat frequency and temperature in the data of all species except *A. mellifera* and *N. testaceicornis*. 

These studies underline the complexity of biological responses to environmental conditions and the limitation of such a general approach. Notably, the correction factor may not uniformly apply across all species, as all species of flying insects do not have the same response to an increase in temperature. Additionally, the general equation obtained in this work was derived from a dataset representative of a specific population of flying insects in New Jersey, raising legitimate questions about its applicability to insect populations in different geographic locations. Nonetheless, optical and acoustic sensors used in entomology often struggle with low taxonomic accuracy and typically categorize a vast number of species into broad clusters. Although there are exceptions, such as bees and bumblebees, our findings indicate a general trend where wingbeat frequencies increase with temperature. Furthermore, this increase accelerates at higher wingbeat frequencies, as modeled by Equation (3) in this study. This insight may enhance the precision of data analysis achievable with optical and acoustic sensors and contributes to ongoing efforts to improve their taxonomic accuracy.

## Figures and Tables

**Table 1:** Average wingbeat frequency and corresponding slope value for five clusters.

Cluster Number	Average Wingbeat Frequency (Hz)	Slope (Hz/°C)
1	50.51	2.02 ± 0.09
2	100.42	3.66 ± 0.15
3	171.33	5.10 ± 0.21
4	290.71	7.69 ± 0.32
5	525.58	9.63 ± 0.40

**Table 2:** Comparison of slopes retrieved in other studies with slopes from the model.

Wingbeat Freq. (Hz)	Slope from Model (Hz/°C)	Slope from Ref. (Hz/°C)	Species Studied in Ref.	Reference
25	0.81	0.6	*Trigona jaty*	Farnworth [66]
96	2.9	0.17	*Popillia japonica*	Ortelli [68]
120	3.57	2.26	*Coleomegilla fuscilabris*	Ortelli [68]
200	5.47	5.4	*Drosophila*	Unwin et al. [67]
240	6.27	6.98	*Periplaneta americana*	Farnworth [66]
400	8.51	8.4	*Ae. aegypti*	Reinhold et al. [71]
400	8.51	8.5	*Ae. aegypti*	Villareal et al. [72]

## Data Availability

The data presented in this study are available on request from the corresponding author.

## References

[1] Wagner D.L., Grames E.M., Forister M.L., Berenbaum M.R., Stopak D. (2021). Insect Decline in the Anthropocene: Death by a Thousand Cuts. Proc. Natl. Acad. Sci. USA.

[2] Buchwald A.G., Hayden M.H., Dadzie S.K., Paull S.H., Carlton E.J. (2020). Aedes-Borne Disease Outbreaks in West Africa: A Call for Enhanced Surveillance. Acta Trop..

[3] Genoud A.P., Saha T., Williams G.M., Thomas B.P. (2023). Insect Biomass Density: Measurement of Seasonal and Daily Variations Using an Entomological Optical Sensor. Appl. Phys. B Lasers Opt..

[4] Saha T., Genoud A.P., Williams G.M., Thomas B.P. (2023). Monitoring the Abundance of Flying Insects and Atmospheric Conditions during a 9-Month Campaign Using an Entomological Optical Sensor. Sci. Rep..

[5] van Klink R., August T., Bas Y., Bodesheim P., Bonn A., Fossøy F., Høye T.T., Jongejans E., Menz M.H.M., Miraldo A. (2022). Emerging Technologies Revolutionise Insect Ecology and Monitoring. Trends Ecol. Evol..

[6] Genoud A.P., Williams G.M., Thomas B.P. (2021). Continuous Monitoring of Aerial Density and Circadian Rhythms of Flying Insects in a Semi-Urban Environment. PLoS ONE.

[7] Genoud A.P., Torsiello J., Belson M., Thomas B.P. (2021). Entomological Photonic Sensors: Estimating Insect Population Density, Its Uncertainty and Temporal Resolution from Transit Data. Ecol. Inform..

[8] Genoud A.P., Gao Y., Williams G.M., Thomas B.P. (2020). A Comparison of Supervised Machine Learning Algorithms for Mosquito Identification from Backscattered Optical Signals. Ecol. Inform..

[9] Genoud A.P., Gao Y., Williams G.M., Thomas B.P. (2019). Identification of Gravid Mosquitoes from Changes in Spectral and Polarimetric Backscatter Cross Sections. J. Biophotonics.

[10] Chatzaki V., Montoro M., El-Rashid R., Jensen A.B., Lecocq A. (2023). A New Approach for Detecting Sublethal Effects of Neonicotinoids on Bumblebees Using Optical Sensor Technology. Insects.

[11] Batista G.E.A.P.A., Hao Y., Keogh E., Mafra-Neto A. Towards Automatic Classification on Flying Insects Using Inexpensive Sensors. Proceedings of the 2011 10th International Conference on Machine Learning and Applications and Workshops (ICMLA 2011).

[12] Naharki K., Huebner C.D., Park Y.-L. (2023). The Detection of Tree of Heaven (*Ailanthus altissima*) Using Drones and Optical Sensors: Implications for the Management of Invasive Plants and Insects. Drones.

[13] Moore A., Miller R.H. (2002). Automated Identification of Optically Sensed Aphid (Homoptera: Aphidae) Wingbeat Waveforms. Ann. Entomol. Soc. Am..

[14] Brydegaard M., Svanberg S. (2018). Photonic Monitoring of Atmospheric and Aquatic Fauna. Laser Photonics Rev..

[15] Kirkeby C., Rydhmer K., Cook S.M., Strand A., Torrance M.T., Swain J.L., Prangsma J., Johnen A., Jensen M., Brydegaard M. (2021). Advances in Automatic Identification of Flying Insects Using Optical Sensors and Machine Learning. Sci. Rep..

[16] Vannoy T.C., Sweeney N.B., Shaw J.A., Whitaker B.M. (2023). Comparison of Supervised Learning and Changepoint Detection for Insect Detection in Lidar Data. Remote Sens..

[17] Kirkeby C., Wellenreuther M., Brydegaard M. (2016). Observations of Movement Dynamics of Flying Insects Using High Resolution Lidar. Sci. Rep..

[18] Fristrup K.M., Shaw J.A., Tauc M.J. (2017). Development of a Wing-Beat-Modulation Scanning Lidar System for Insect Studies. Lidar Remote Sensing for Environmental Monitoring 2017.

[19] Li Y., Wang K., Quintero-Torres R., Brick R., Sokolov A.V., Scully M.O. (2020). Insect Flight Velocity Measurement with a CW Near-IR Scheimpflug Lidar System. Opt. Express.

[20] Rigakis I., Potamitis I., Tatlas N.A., Livadaras I., Ntalampiras S. (2019). A Multispectral Backscattered Light Recorder of Insects’ Wingbeats. Electronics.

[21] Zhu S., Malmqvist E., Li W., Jansson S., Li Y., Duan Z., Svanberg K., Feng H., Song Z., Zhao G. (2017). Insect Abundance over Chinese Rice Fields in Relation to Environmental Parameters, Studied with a Polarization-Sensitive CW near-IR Lidar System. Appl. Phys. B Lasers Opt..

[22] Hao Z., Drake V.A., Taylor J.R., Warrant E. (2020). Insect Target Classes Discerned from Entomological Radar Data. Remote Sens..

[23] Brydegaard M., Jansson S. (2019). Advances in Entomological Laser Radar. J. Eng..

[24] Wang R., Hu C., Fu X., Long T., Zeng T. (2017). Micro-Doppler Measurement of Insect Wing-Beat Frequencies with W-Band Coherent Radar. Sci. Rep..

[25] Zhang T., Liu X., Hu C., Wang R., Liu C., Li W. (2019). Insect Wing-beat Frequency Automatic Extraction and Experimental Verification with a Ku-band Insect Radar System. J. Eng..

[26] Chapman J.W., Drake V.A., Reynolds D.R. (2011). Recent Insights from Radar Studies of Insect Flight. Annu. Rev. Entomol..

[27] Noskov A., Bendix J., Friess N. (2021). A Review of Insect Monitoring Approaches with Special Reference to Radar Techniques. Sensors.

[28] Bauer S., Chapman J.W., Reynolds D.R., Alves J.A., Dokter A.M., Menz M.M.H., Sapir N., Ciach M., Pettersson L.B., Kelly J.F. (2017). From Agricultural Benefits to Aviation Safety: Realizing the Potential of Continent-Wide Radar Networks. Bioscience.

[29] Osborne J.L., Clark S.J., Morris R.J., Williams I.H., Riley J.R., Smith A.D., Reynolds D.R., Edwards A.S. (1999). A Landscape-Scale Study of Bumble Bee Foraging Range and Constancy, Using Harmonic Radar. J. Appl. Ecol..

[30] Maggiora R., Saccani M., Milanesio D., Porporato M. (2019). An Innovative Harmonic Radar to Track Flying Insects: The Case of Vespa Velutina. Sci. Rep..

[31] Lavrenko A., Barry Z., Norman R., Frazer C., Ma Y., Woodward G., Pawson S. Autonomous Swarm of UAVs for Tracking of Flying Insects with Harmonic Radar. Proceedings of the 2021 IEEE 93rd Vehicular Technology Conference (VTC2021-Spring).

[32] González-Pérez M.I., Faulhaber B., Williams M., Brosa J., Aranda C., Pujol N., Verdún M., Villalonga P., Encarnação J., Busquets N. (2022). A Novel Optical Sensor System for the Automatic Classification of Mosquitoes by Genus and Sex with High Levels of Accuracy. Parasit. Vectors.

[33] Chen Y., Why A., Batista G., Mafra-Neto A., Keogh E. (2014). Flying Insect Classification with Inexpensive Sensors. J. Insect Behav..

[34] Li Y., Kiskin I., Sinka M., Zilli D., Chan H., Herreros-Moya E., Chareonviriyaphap T., Tisgratog R., Willis K., Roberts S. Fast Mosquito Acoustic Detection with Field Cup Recordings: An Initial Investigation. Proceedings of the Detection and Classification of Acoustic Scenes and Events 2018.

[35] Manoukis N.C., Collier T.C. (2019). Computer Vision to Enhance Behavioral Research on Insects. Ann. Entomol. Soc. Am..

[36] Lima M.C.F., de Almeida Leandro M.E.D., Valero C., Coronel L.C.P., Bazzo C.O.G. (2020). Automatic Detection and Monitoring of Insect Pests—A Review. Agriculture.

[37] Preti M., Verheggen F., Angeli S. (2021). Insect Pest Monitoring with Camera-Equipped Traps: Strengths and Limitations. J. Pest Sci..

[38] Johnson B.J., Manby R., Devine G.J. (2022). The Use of Automated Traps to Assess the Efficacy of Insecticide Barrier Treatments Against Abundant Mosquitoes in Remote Environments. J. Med. Entomol..

[39] Santoso G., Hani S., Kabalmay H.K., Mubarok I.A., Susetyo J. (2023). Insects Pest Trap Monitoring System Using Internet of Things Based Sensors. Eng. Technol. J..

[40] Voloshin V., Kröner C., Seniya C., Murray G.P.D., Guy A., Towers C.E., McCall P.J., Towers D.P. (2020). Diffuse Retro-Reflective Imaging for Improved Video Tracking of Mosquitoes at Human Baited Bednets. R. Soc. Open Sci..

[41] Rhodes M.W., Bennie J.J., Spalding A., Ffrench-Constant R.H., Maclean I.M.D. (2022). Recent Advances in the Remote Sensing of Insects. Biol. Rev..

[42] Wang Y., Zhao C., Dong D., Wang K. (2023). Real-Time Monitoring of Insects Based on Laser Remote Sensing. Ecol. Indic..

[43] Mullen E.R., Rutschman P., Pegram N., Patt J.M., Adamczyk J.J. (2016). Johanson Laser System for Identification, Tracking, and Control of Flying Insects. Opt. Express.

[44] Keller M.D., Norton B.J., Farrar D.J., Rutschman P., Marvit M., Makagon A. (2020). Optical Tracking and Laser-Induced Mortality of Insects during Flight. Sci. Rep..

[45] Gaetani R., Lacotte V., Dufour V., Clavel A., Duport G., Gaget K., Calevro F., Da Silva P., Heddi A., Vincent D. (2021). Sustainable Laser-Based Technology for Insect Pest Control. Sci. Rep..

[46] Lacotte V., NGuyen T., Sempere J.D., Novales V., Dufour V., Moreau R., Pham M.T., Rabenorosoa K., Peignier S., Feugier F.G. (2022). Pesticide-Free Robotic Control of Aphids as Crop Pests. AgriEngineering.

[47] Hu P.-S., Chou C.-C., Huang C.-G., Tu W.-C., Wang H.-Y., Chan M.-C. (2019). Knocking down Free-Flight Adult Mosquitoes via Dynamic Tracking. OSA Contin..

[48] Rakhmatulin I. (2021). Raspberry PI for Kill Mosquitoes by Laser. Preprints.

[49] Rakhmatulin I., Lihoreau M., Pueyo J. (2023). Selective Neutralisation and Deterring of Cockroaches with Laser Automated by Machine Vision. Orient. Insects.

[50] Zaidem A., Silva L., Ferreira A., Carvalho M., Ragni M., Abegão L., Pinheiro P. (2023). New Biocompatible Technique Based on the Use of a Laser to Control the Whitefly Bemisia Tabaci. Photonics.

[51] Patt J.M., Makagon A., Norton B., Marvit M., Rutschman P., Neligeorge M., Salesin J. (2024). An Optical System to Detect, Surveil, and Kill Flying Insect Vectors of Human and Crop Pathogens. Sci. Rep..

[52] Genoud A.P., Basistyy R., Williams G.M., Thomas B.P. (2018). Analysis of Predictor Variables for Mosquito Species Identification from Dual-Wavelength Polarization-Sensitive Lidar Measurements. Lidar Remote Sensing for Environmental Monitoring XVI.

[53] Kittichai V., Pengsakul T., Chumchuen K., Samung Y., Sriwichai P., Phatthamolrat N., Tongloy T., Jaksukam K., Chuwongin S., Boonsang S. (2021). Deep Learning Approaches for Challenging Species and Gender Identification of Mosquito Vectors. Sci. Rep..

[54] Rydhmer K., Bick E., Still L., Strand A., Luciano R., Helmreich S., Beck B.D., Grønne C., Malmros L., Poulsen K. (2022). Automating Insect Monitoring Using Unsupervised Near-Infrared Sensors. Sci. Rep..

[55] Siddiqui A.A., Kayte D.C. Convolution Neural Network-Based Mosquito Classification System. Proceedings of the 3rd International Conference on ICT for Digital, Smart, and Sustainable Development, ICIDSSD 2022.

[56] Taylor L.R. (1963). Analysis of the Effect of Temperature on Insects in Flight. J. Anim. Ecol..

[57] Spangler H.G., Buchmann S.L. (2014). Effects of Temperature on Wingbeat Frequency in the Solitary Bee Centris Caesalpiniae (Anthophoridae: Hymenoptera). J. Kansas Entomol. Soc..

[58] Church N.S. (1960). Heat Loss and the Body Temperatures of Flying Insects. J. Exp. Biol..

[59] Yu W., Zhang H., Xu R., Sun Y., Wu K. (2022). Characterization of Wingbeat Frequency of Different Taxa of Migratory Insects in Northeast Asia. Insects.

[60] Foster J.A., Robertson M. (2016). Temperature Dependency of Wing-Beat Frequency in Intact and Deafferented Locustus. J. Exp. Biol..

[61] Pinto J., Magni P.A., O’Brien R.C., Dadour I.R. (2022). Chasing Flies: The Use of Wingbeat Frequency as a Communication Cue in Calyptrate Flies (Diptera: Calyptratae). Insects.

[62] Sotavalta O. (1952). Flight-Tone and Wing-Stroke Frequency of Insects and the Dynamics of Insect Flight. Nature.

[63] Gilmour K.M., Ellington C.P. (1993). Power Output of Glycerinated Bumblebee Flight Muscle. J. Exp. Biol..

[64] Rowley W.A., Graham C.L. (1968). The Effect of Temperature and Relative Humidity on the Flight Performance of Female Aedes Aegypti. J. Insect Physiol..

[65] Mahmood F., Crans W.J. (1998). Effect of Temperature on the Development of Culiseta Melanura (Diptera: Culicidae) and Its Impact on the Amplification of Eastern Equine Encephalomyelitis Virus in Birds. J. Med. Entomol..

[66] Farnworth E.G. (1972). Effects of Ambient Temperature, Humidity, and Age on Wing-Beat Frequency of Periplaneta Species. J. Insect Physiol..

[67] Unwin D.M., Corbet S.A. (1984). Wingbeat Frequency, Temperature and Body Size in Bees and Flies. Physiol. Entomol..

[68] Oertli J.J. (1989). Relationship of Wing Beat Frequency and Temperature During Take-Off Flight in Temperate-Zone Beetles. J. Exp. Biol..

[69] Huang J., Zhang G., Wang Y. (2013). Effects of Age, Ambient Temperature and Reproductive Status on Wing Beat Frequency of the Rice Leafroller Cnaphalocrocis Medinalis (Guenée) (Lepidoptera: Crambidae). Appl. Entomol. Zool..

[70] Satopää V., Albrecht J., Irwin D., Raghavan B. Finding a “Kneedle” in a Haystack: Detecting Knee Points in System Behavior. Proceedings of the 2011 31st International Conference on Distributed Computing Systems Workshops.

[71] Reinhold J.M., Lazzari C.R., Lahondère C. (2018). Effects of the Environmental Temperature on Aedes Aegypti and Aedes Albopictus Mosquitoes: A Review. Insects.

[72] Villarreal S.M., Winokur O., Harrington L. (2017). The Impact of Temperature and Body Size on Fundamental Flight Tone Variation in the Mosquito Vector Aedes Aegypti (Diptera: Culicidae): Implications for Acoustic Lures. J. Med. Entomol..

[73] Sotavalta O. (1963). The Flight Sounds of Insects.

[74] Parmezan A.R.S., Souza V.M.A., Žliobaitė I., Batista G.E.A.P.A. (2021). Changes in the Wing-Beat Frequency of Bees and Wasps Depending on Environmental Conditions: A Study with Optical Sensors. Apidologie.

